# Identification of a *GNE* homozygous mutation in a Han‐Chinese family with GNE myopathy

**DOI:** 10.1111/jcmm.13827

**Published:** 2018-08-29

**Authors:** Yuan Wu, Lamei Yuan, Yi Guo, Anjie Lu, Wen Zheng, Hongbo Xu, Yan Yang, Pengzhi Hu, Shaojuan Gu, Bingqi Wang, Hao Deng

**Affiliations:** ^1^ Center for Experimental Medicine The Third Xiangya Hospital Central South University Changsha China; ^2^ Department of Clinical Laboratory The Third Xiangya Hospital Central South University Changsha China; ^3^ Department of Medical Information Information Security and Big Data Research Institute Central South University Changsha China; ^4^ Department of Orthopedics The Third Xiangya Hospital Central South University Changsha China; ^5^ Department of Neurology The Third Xiangya Hospital Central South University Changsha China; ^6^ Department of Radiology The Third Xiangya Hospital Central South University Changsha China

**Keywords:** GNE myopathy, homozygous, missense mutation, the *GNE* gene

## Abstract

GNE myopathy is a rare, recessively inherited, early adult‐onset myopathy, characterized by distal and proximal muscle degeneration which often spares the quadriceps. It is caused by mutations in the UDP‐N‐acetylglucosamine 2‐epimerase/N‐acetylmannosamine kinase gene (*GNE*). This study aimed to identify the disease‐causing mutation in a three‐generation Han‐Chinese family with members who have been diagnosed with myopathy. A homozygous missense mutation, c.1627G>A (p.V543M) in the *GNE* gene co‐segregates with the myopathy present in this family. A GNE myopathy diagnosis is evidenced by characteristic clinical manifestations, rimmed vacuoles in muscle biopsies and the presence of biallelic *GNE* mutations. This finding broadens the *GNE* gene mutation spectrum and extends the GNE myopathy phenotype spectrum.

## 1 INTRODUCTION

GNE myopathy, also referred to as Nonoaka myopathy, was first described as distal myopathy having rimmed vacuoles (DMRV) in 1981 in Japanese patients, later called “hereditary inclusion body myopathy (HIBM)” or “inclusion body myopathy 2 (IBM2)”. It is a rare, recessively inherited, early adult‐onset myopathy.^1^ It is typically characterized by slow progression that preferentially affects the tibialis anterior muscles, commonly spares the quadriceps femoris muscles and gradually spreads to other muscles.^2^, ^3^ GNE myopathy occurs worldwide. The prevalence ranges from 1/1 000 000 to 21/1 000 000, although it may be underestimated as many patients escape diagnosis.^1^, ^4^ It has a higher prevalence in Middle Eastern Jews and Japanese. GNE myopathy is caused by the mutations in the UDP‐N‐acetylglucosamine 2‐epimerase/N‐acetylmannosamine kinase gene (*GNE*), which encodes the bifunctional enzyme catalysing the first two rate‐limiting steps in sialic acid biosynthesis.^5^

In this study, targeted exome capture and high‐throughput sequencing of 411 known genes involved in neuromuscular disorders were performed to identify genetic causes of an autosomal recessive GNE myopathy in a three‐generation Han‐Chinese pedigree.^6^ A homozygous missense mutation, c.1627G>A (p.V543M), in the *GNE* gene was found to co‐segregate with the GNE myopathy present in this family.

## 2 MATERIALS AND METHODS

### 2.1 Participators and clinical evaluation

**Figure 1:**
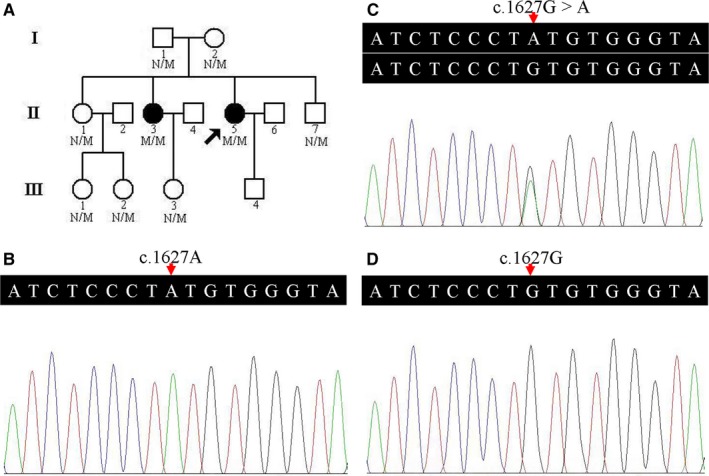
Pedigree data of a GNE myopathy family. A, Pedigree of the family with GNE myopathy. N: normal; M: the *GNE* c.1627G>A (p.V543M) mutation. Arrow indicates the proband. B, Sequence of homozygous c.1627G>A (p.V543M) variant. C, Sequence of heterozygous c.1627G>A (p.V543M) variant. D, Sequence of a normal control

A 13‐person, three‐generation Han‐Chinese pedigree was recruited at the Third Xiangya Hospital, Central South University, Changsha, Hunan, China (Figure 1A). Clinical data, and peripheral blood samples were obtained from 9 members, including 2 affected (II:3 and II:5) and 7 unaffected individuals (I:1, I:2, II:1, II:7, III:1, III:2 and III:3). Blood samples were collected from 200 unrelated, ethnically matched normal controls (male/female: 100/100, age 38.5 ± 5.6 years). Written informed consents were obtained from participants or their guardians. The study complied with the Declaration of Helsinki Principles and was approved by the Ethics Committee of the Third Xiangya Hospital of Central South University in Changsha, Hunan, China.

Two patients (II:3 and II:5) underwent detailed clinical evaluation, including onset age and symptoms, muscle weakness distribution, electrocardiography (ECG), serum creatine kinase (CK), electromyography (EMG) and magnetic resonance imaging (MRI) of thigh, leg and buttock muscles. Muscle biopsy was obtained from the proband (II:5) left biceps.

### 2.2 Variant analysis and direct Sanger sequencing

Genomic DNA (gDNA) was isolated from peripheral blood using the phenol‐chloroform extraction method.^7^, ^8^, ^9^, ^10^ A targeted deep sequencing analysis in the proband was performed. A panel of 411 genes including known neuromuscular disorder‐related genes was captured using OncoCap Enrichment System (MyGenostics, Beijing, China) via reported methods.^11^ After enrichment, libraries were sequenced using an Illumina Solexa HiSeq 2000 sequencer.^11^, ^12^ Filtrations of all identified variations were performed based on following references: mean coverage ≥100, mutation ratio ≥10, absence of mutation in 1000 Genomes Project and non‐synonymous mutations.^13^, ^14^ Direct Sanger sequencing confirmed the potential pathogenic variant via an ABI3500 sequencer (Applied Biosystems, Foster City, CA, USA).^15^, ^16^, ^17^ PCR amplification and Sanger sequencing primer sequences are as follows: 5′‐CCTTGTGTTTGTGGTGGAGC‐3′ and 5′‐CCTCTTACCTGTGCCTGTGA‐3′.

### 2.3 Bioinformatics analysis of the mutation

Multiple sequence alignments and conservation analysis were conducted using the Basic Local Alignment Search Tool (http://blast.st-va.ncbi.nlm.nih.gov/Blast.cgi) across various species, including Homo sapiens, Pan troglodytes, Macaca mulatta, Canis lupus familiaris, Bos taurus, Mus musculus, Rattus norvegicus, Gallus gallus and Danio rerio.^13^ Functional prediction software programs, such as polymorphism Phenotyping version 2 (PolyPhen‐2, http://genetics.bwh.harvard.edu/pph2/), Sorting Intolerant From Tolerant (SIFT, http://sift.jcvi.org/), MutPred2 (http://mutpred.mutdb.org/) and MutationTaster (http://www.mutationtaster.org/), were used to predict the possible effects of the identified alteration on protein structure and function.^1^

## 3 RESULTS

### 3.1 Pedigree clinical characteristics

**Figure 2:**
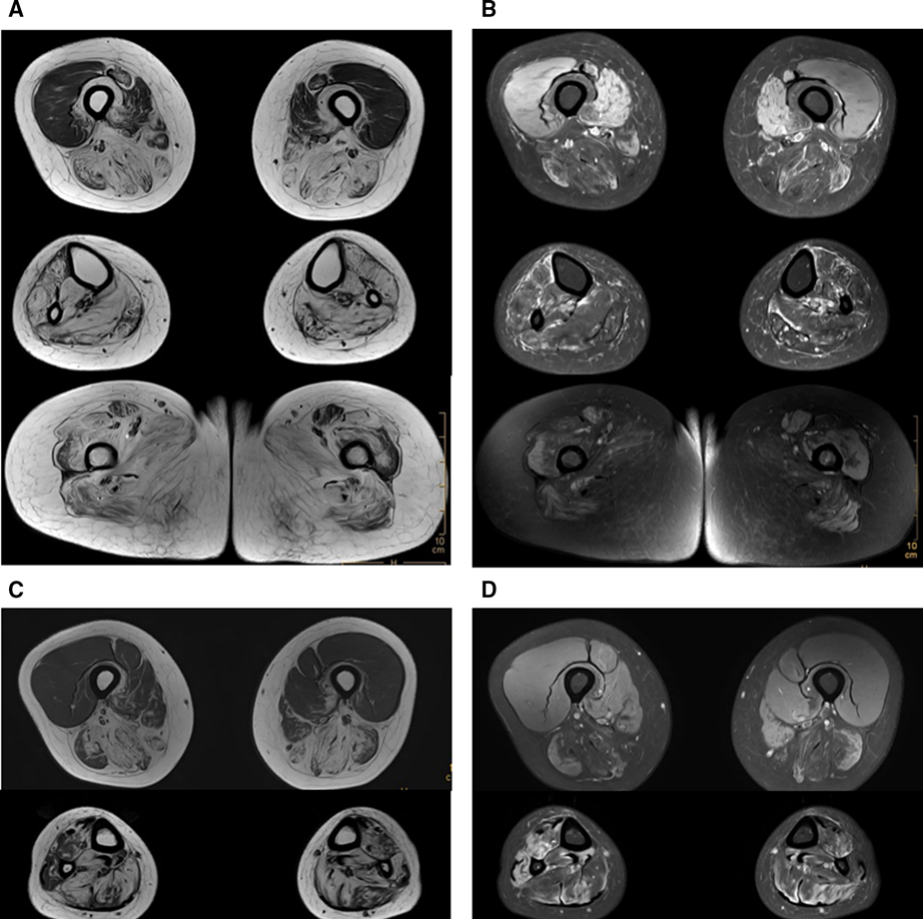
MRI in patients (II:3 and II:5) revealed serious fatty replacement in posterior and medial compartments of the thigh muscles and almost all lower leg muscles, which also presented in the buttock muscles of the patient (II:3). (A and B), axial T1‐weighted and axial PDw SPAIR MRI of thigh, lower leg and buttock muscles in patient (II:3). (C and D), axial T1‐weighted and axial PDw SPAIR MRI of thigh and lower leg muscles in patient (II:5)

Tibialis anterior muscles weakness onset began in the proband (II:5) and her elder affected sister (II:3) at age of 26 and 24, respectively. They subsequently had difficulty climbing stairs and walking. Both now have a “duck” gait. Neurological evaluations revealed biceps, triceps, hip adductors, hip abductors and knee flexors were seriously affected. Hip‐girdles were involved to a lesser degree. Wrist flexors, extensors and interossei muscles were nearly normal. ECG findings were within normal ranges. Proband left bicep muscle biopsies were performed. Pathological features showed a pattern of muscular dystrophy with rimmed vacuoles present in the muscle fibres as well as fibre diameter variations, but without evidence of inflammation. Neither necrosis nor regeneration of muscle fibres was observed. Electron microscopy revealed fibre size variations. CK levels were 310 U/L and 287 U/L, which are moderately elevated above the normal values of 40–200 U/L (Female). EMG analysis showed normal nerve conduction velocities, and myopathic damage was observed in almost all tested muscles of the two patients. Muscle MRI examination of legs showed predominantly fatty changes in posterior and medial compartments of the thigh, and almost all lower leg muscles in both patients, and the buttock muscles of patient (II:3) showed significant fatty replacement (Figure 2). Pedigree clinical characteristics appear in Table 1.

**Table 1:** Clinical characteristics of affected family members with GNE myopathy

	II:3	II:5
Age (years)	32	30
Age at onset of symptoms (years)	24	26
Sex	Female	Female
Genotype	Homozygote	Homozygote
Onset symptoms	Difficulty in climbing stairs	Difficulty in climbing stairs
Walking capability	Ambulant with assistance	Ambulant with assistance
ECG	Normal	Normal
EMG	Normal NCV, myogenic damage	Normal NCV, myogenic damage
Serum CK levels (U/L)^a^	287	310
Muscle strength (MRC grade; Left/Right)		
Biceps	2/2	3+/3+
Triceps	2/2	3+/3+
Wrist flexor	4/4	4+/4+
Wrist extensor	4/4	4+/4+
Interossei	4+/4+	4+/4+
Hip‐girdles	3/4	3/4
Hip adductors	1/1+	1/1+
Hip abductors	1/2	1+/2+
Knee flexors	1+/1+	2/3

ECG, electrocardiography; EMG, electromyography; NCV, nerve conduction velocities; CK, serum creatine kinase.

a Normal range: 40‐200 U/L (female), MRC: Medical Research Council.

Family members denied consanguineous marriage. Sibling disease co‐occurrence and the absence of transmission through different generations indicate an autosomal recessive inheritance mode.

### 3.2 *GNE* mutation screening

A prioritization scheme similar to those described in recent studies was used to identify proband pathogenic variants.^13^, ^18^, ^19^ Only a homozygous missense variant, c.1627G>A (p.V543M), in the *GNE* gene (NM_001128227, hGNE2 isoform NP_001121699) was suspected as the proband pathogenic variant. No other variants of known disease‐causing genes of neuromuscular disorders were identified. The variant was subsequently confirmed by Sanger sequencing. The same homozygous variant was identified in her affected elder sister (II:3) (Figure 1B). Family members (I:1, I:2, II:1, II:7, III:1, III:2 and III:3) presented heterozygous variant (Figure 1C). The homozygous variant co‐segregated with disease in the family, and this variant was absent from both the 200 unrelated Han‐Chinese healthy controls and the public databases, such as 1000 Genomes Project, Exome Aggregation Consortium database (ExAC), NHLBI GO Exome Sequencing Project (ESP) and Genome Aggregation Database (gnomAD) (Figure 1D).

### 3.3 Bioinformatics analysis of the mutation

**Figure 3:**
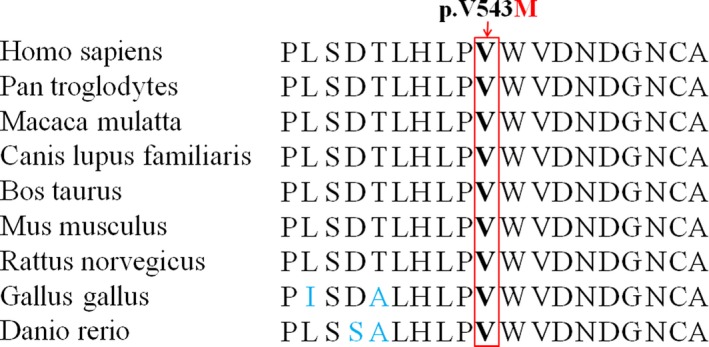
Conservation analysis of the GNE p.Val543 amino acid residue

Valine at position 543 (p.Val543) is conserved across different species (Figure 3). Based on the predicted values of several functional prediction software programs, including PolyPhen‐2, SIFT, MutPred2 and MutationTaster, c.1627G>A (p.V543M) in the *GNE* gene is deemed “severe”, that is protein damaging. The predicted scores and results of functional prediction software programs appear in Table 2.

**Table 2:** The predicted results of c.1627G>A (p.V543M) in the *GNE* gene from several functional prediction software programs

Software	Score	Prediction
SIFT	0.01	Damaging
PolyPhen‐2	0.952 (sensitivity, 0.64; specificity, 0.92)	Probably damaging
MutPred2	0.861 (>0.75)	Highly harmful
MutationTaster	0.999	Disease causing

## 4 DISCUSSION

The *GNE* gene, mapped to chromosome 9p13.3, spans 62.6 kb and contains 13 exons.^1^ Eight different *GNE* mRNA splice variants encode multiple protein isoforms, the longest consists of 753 amino acids (NM_001128227, NP_001121699, all mutations in this study are described based upon this transcript).^20^, ^21^ The GNE protein contains two functional domains: an N‐terminal epimerase domain and a C‐terminal kinase domain.^20^ It plays a key role in the biosynthesis of N‐acetylneuraminic acid, a member of the sialic acid family, which is an important component of cell surface glycoproteins, glycolipids, polysaccharides and gangliosides. These play important roles in cell adhesion and signal transduction.^22^ The GNE protein ubiquitously expresses in almost all tissues,^20^ with the highest levels in liver and placenta, while relatively low levels in skeletal muscle. It localizes to the cytoplasm, the Golgi‐region and the cell nucleus.^1^, ^23^

Non‐allosteric, homozygous or compound heterozygous, primarily missense mutations in the *GNE* gene result in reduced GNE epimerase and kinase enzymatic activity. This leads to decreased sialic acid production, which appears to be the main contributor to this still elusive disease pathology.^1^ Normal sialylation is crucial to skeletal muscle glycoprotein function. Abnormal glycoprotein sialylation in cell surfaces may interfere with cell adhesion and signal transduction, and trigger myofibrillar degeneration. This results in loss of normal muscle function. Some skeletal muscle proteins and neural cell adhesion molecule are hyposialylated in GNE myopathy. This is a distinctive feature compared to other myopathies with similar clinical manifestations. However, the effect of *GNE* variants on sialylation is unsettled and has yet to be satisfactorily elucidated.^20^

In mice, GNE protein expresses at an early embryonic stage and *Gne* ^−/−^ mice is lethal.^4^, ^24^ This is consistent with the lack of biallelic null mutations and only “mildly deleterious” mutations reported in GNE myopathy patients.^4^ Given that *Gne*‐deficient hyposialylation is the core pathogenic factor, experimental prophylactic treatments with N‐acetylmannosamine or sialic acid metabolites were performed, and effectively avoiding muscle weakness and atrophy in *Gne*‐deficient mice models were found.^25^, ^26^, ^27^, ^28^ This shed a new light on targeted therapy for disease in humans in the near future.

To date, more than 201 *GNE* mutations across the entire coding region have been reported. They span the epimerase and kinase domains, and allosteric region.^2^, ^5^ Middle Eastern mutation c.2228T>C (p.M743T) appears predominantly in those of Middle Eastern population, while c.1807G>C (p.V603L) and c.620A>T (p.D207V) are most common in Japanese patients.^29^, ^30^

This study identifies a homozygous missense mutation, c.1627G>A (p.V543M), in a Han‐Chinese family. This mutation is previously reported as a mutant allele (originally termed c.1534G>A, p.V512M, NM_005476) of a compound heterozygote (p.V543M and p.G579S) in a Chinese patient.^2^ It is located in the kinase domain, which may cause a severe phenotype compared to those mapped in other regions. The patients here presented well‐known typical clinical features of GNE myopathy, including progressive distal muscular atrophy and weakness. These are consistent with the reports of prior studies.^2^, ^5^ It is distinguishable from atypical patients who had at least one allele mutation in the non‐kinase domain.^2^, ^5^, ^31^ The presence of different genetic backgrounds, epigenetic factors and/or modifying environmental events may contribute significantly to the clinical manifestations.^20^ Parental consanguinity was denied by the family, but the mutant allele may originate from a founder as the patients’ parents are from the same geographical region.

## 5 CONCLUSIONS

In summary, a homozygous missense mutation c.1627G>A (p.V543M) in the *GNE* gene was identified in a Han‐Chinese family. The characteristic clinical manifestations, rimmed vacuoles in muscle biopsies and identification of biallelic *GNE* mutations, supported a diagnosis of GNE myopathy.^2^, ^4^ To our knowledge, this is the first report of the homozygous c.1627G>A mutation in the *GNE* gene in patients. Further functional studies and in vitro and/or in vivo models with genetic deficiencies, especially mutant knock‐in models are warranted. Future therapeutic strategies might, among other things, focus on metabolic supplementation, better GNE metabolites or sialic acid compounds, medications to block or modify degenerative process, gene or cell‐based therapies and the AAV‐mediated gene vector for systemic administration of *GNE*.^4^

## COMPLIANCE WITH ETHICAL STANDARDS

Informed consent was obtained from the individuals. The study received approval from the Ethics Committee of the Third Xiangya Hospital, Central South University, Changsha, Hunan, China.

## CONFLICT OF INTEREST

The authors declare that they have no conflict of interest.
